# Anterior cingulate cortex in complex associative learning: monitoring action state and action content

**DOI:** 10.1101/2025.01.29.635442

**Published:** 2025-01-29

**Authors:** Wenqiang Huang, Arron F Hall, Natalia Kawalec, Ashley N Opalka, Jun Liu, Dong V Wang

**Affiliations:** 1 Department of Neurobiology & Anatomy, Drexel University College of Medicine, Philadelphia, PA 19129.; 2 School of Arts & Sciences, University of Pennsylvania, Philadelphia, PA 19104.

## Abstract

Environmental changes necessitate adaptive responses, and thus the ability to monitor one’s actions and their connection to specific cues and outcomes is crucial for survival. The anterior cingulate cortex (ACC) is implicated in these processes, yet its precise role in action monitoring and outcome evaluation remains unclear. To investigate this, we developed a novel discrimination–avoidance task for mice, designed with clear temporal separation between actions and outcomes. Our findings show that ACC neurons primarily encode post-action variables over extended periods, reflecting the animal’s preceding actions rather than the outcomes or values of those actions. Specifically, we identified two distinct subpopulations of ACC neurons: one encoding the action state (whether an action was taken) and the other encoding the action content (which action was taken). Importantly, increased post-action ACC activity was associated with better performance in subsequent trials. These findings suggest that the ACC supports complex associative learning through extended signaling of rich action-relevant information, thereby bridging cue, action, and outcome associations.

## Introduction

The ability to adapt behavior based on environmental cues and outcome-related feedback is essential for survival. Central to this process is the brain’s capacity to integrate various types of information to form cue–action–outcome associations that guide future behaviors. However, the mechanisms which enable the brain to accomplish such complex cognitive control remains unclear. The anterior cingulate cortex (ACC) has emerged as a critical node in mediating this process, with growing evidence underscoring its pivotal role in cognitive flexibility and adaptive behavior [1–7]. Despite this, the exact function of the ACC and its role in updating and modifying behavior remain subjects of ongoing debate [1–7].

Historically, the ACC was thought to be essential for error or conflict monitoring [8–10]. However, these views have been challenged by recent findings. Specifically, ACC activity does not merely track errors, as it can be driven by contexts in which errors are likely to, but do not actually, occur [11, 12]. Moreover, ACC activity can be triggered by stimuli signaling surprisingness [13, 14], or the necessity of strategy shift regardless of error commission [15]. Similarly, ACC neuronal activity does not appear to directly monitor conflicts, including conflicting visual and spatial cues [13, 16–18]. Instead, recent studies suggest that ACC activity is primarily associated with actions and/or action-associated outcomes, especially when the task involves competing action selections [14, 19–27]. Supporting this, studies have shown that lesions of the ACC impair the maintenance of newly acquired task performance as well as the ability to link actions with their associated outcomes [27, 28].

Despite evidence linking the ACC to action-related processes, its specific role in naturalistic behaviors – those extending beyond basic motor tasks such as licking, making a saccade, pressing a lever, or moving a joystick – remains poorly understood. Additionally, the often lack of clear temporal boundaries between actions and outcomes in prior studies prevents definitive conclusions regarding whether ACC neurons are involved in extended monitoring of actions, or solely in tracking action-associated outcomes. In this study, we aim to disentangle the ACC’s role by employing a novel behavioral task designed to evoke robust naturalistic action responses and establish a clear temporal separation between actions and their corresponding outcomes. Our findings reveal a distinct role of the ACC in encoding post-action variables that reflect animals’ preceding actions rather than the outcomes or values of those actions.

## Results

### A novel discrimination–avoidance task

**Figure 1.**
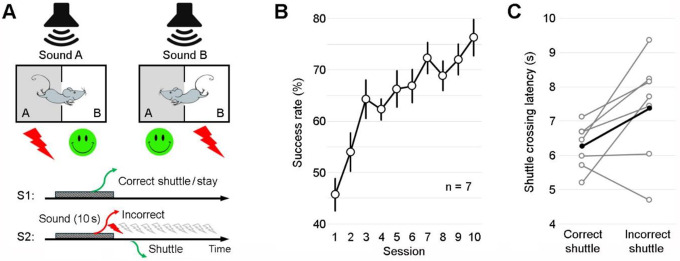
A novel discrimination–avoidance task. **A**, Top: Schematic of the task. Mice are trained to discriminate between a pair of sounds (lasting 10 s) and shuttle between two distinct rooms of a shuttle box to avoid footshocks. Specifically, sounds A and B signal shocks in rooms A and B, respectively. Bottom: Two behavioral response scenarios. S1: If the mouse makes the correct response, either by staying in or shuttling to the correct room before the sound ends, no shock is administered. S2: If the mouse makes an incorrect response, either by staying in or shuttling to the incorrect room before the sound ends, up to 10 mild shocks are administered until the mouse shuttles to the correct room. Each training session comprises 50 trials, 60 s apart; sounds A and B are presented in a pseudorandom order. **B**, Learning curve showing the success rate in avoiding shocks across training sessions (mean ± s.e.m.; n = 7). One-way ANOVA: F_9, 60_ = 8.04; P = 1.07 × 10^−7^. **C**, The mean shuttle crossing latencies for correct and incorrect shuttles (averaged over the last two sessions) show a trending significant difference (P = 0.074, paired *t* test). The black line indicates the mean; grey lines indicate individual mice. Shuttle crossing is defined as the body center crossing the midline opening of the shuttle box.

We first developed a novel discrimination–avoidance task, tailored to investigate action-based complex associative learning in mice. In this task, animals learn to discriminate between a pair of sounds that predict context-dependent footshocks between two visually distinct compartments. Specifically, sounds A and B signal electric shocks in rooms A and B of a shuttle box at sound terminations, respectively (Fig. 1A; Supplementary Fig. 1). This prompts the animals to either “stay” in the current room or “shuttle” to the other room during sound presentations to avoid shocks. Notably, the two sounds are dynamically linked to safety or threat depending on the mouse’s location during the sound presentation. This task, which requires the discrimination of sensory cues and environmental contexts, as well as the integration of cues, actions, and outcomes, serves as an ideal tool to study complex associative learning.

Our results show that mice gradually learn the task, achieving a success rate of 76.3% on average in avoiding shocks by the end of training (Fig. 1B), with the top performer reaching a success rate of 94%. On average, the shuttle response latency is 6.3 s during correct shuttles, leaving a 3.7-s temporal separation between actions (shuttles) and outcomes (safety or shocks; Fig. 1C). This clear temporal distinction allows us to compare how information is coded during the action vs. outcome periods. The slightly longer shuttle latency during incorrect shuttles suggests that last-second responses are more likely to be incorrect (Fig. 1C).

### ACC neurons exhibit robust post-action firing changes

**Figure 2.**
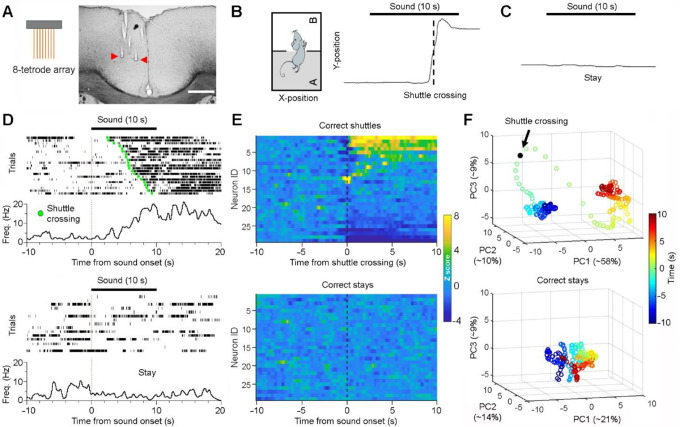
ACC neurons primarily encode post-action variables. **A**, Schematic of an 8-tetrode array and a representative brain section highlighting the recording sites (red arrows) within the ACC. Scale bar, 0.5 mm. **B**, Diagrams showing a representative shuttle route (left) and corresponding Y-position of the animal’s body center (right). A shuttle response typically lasts ~1–3 s (Supplementary Fig. 2). **C**, An example Y-position of the animal’s body center during a stay trial. **D**, Peri-event rasters and histograms of an ACC neuron during correct-shuttle trials (top; arranged based on shuttle latencies) and correct-stay trials (bottom). **E**, A representative heatmap activity of simultaneously recorded ACC neurons (n = 29) during correct shuttles (top) and correct stays (bottom) from the same recording session. **F**, Principal component analysis (PCA) of ACC neuronal population activity as shown in E. PC1, PC2, and PC3 are the first three principal components; the numbers are the percentages of total variance explained by the corresponding PCs. Each circle indicates a time lapse of 0.1 s. Note that there is a robust neural state change in the 3-D PC space surrounding the shuttle response (top), but not the stay response (bottom).

**Figure 3.**
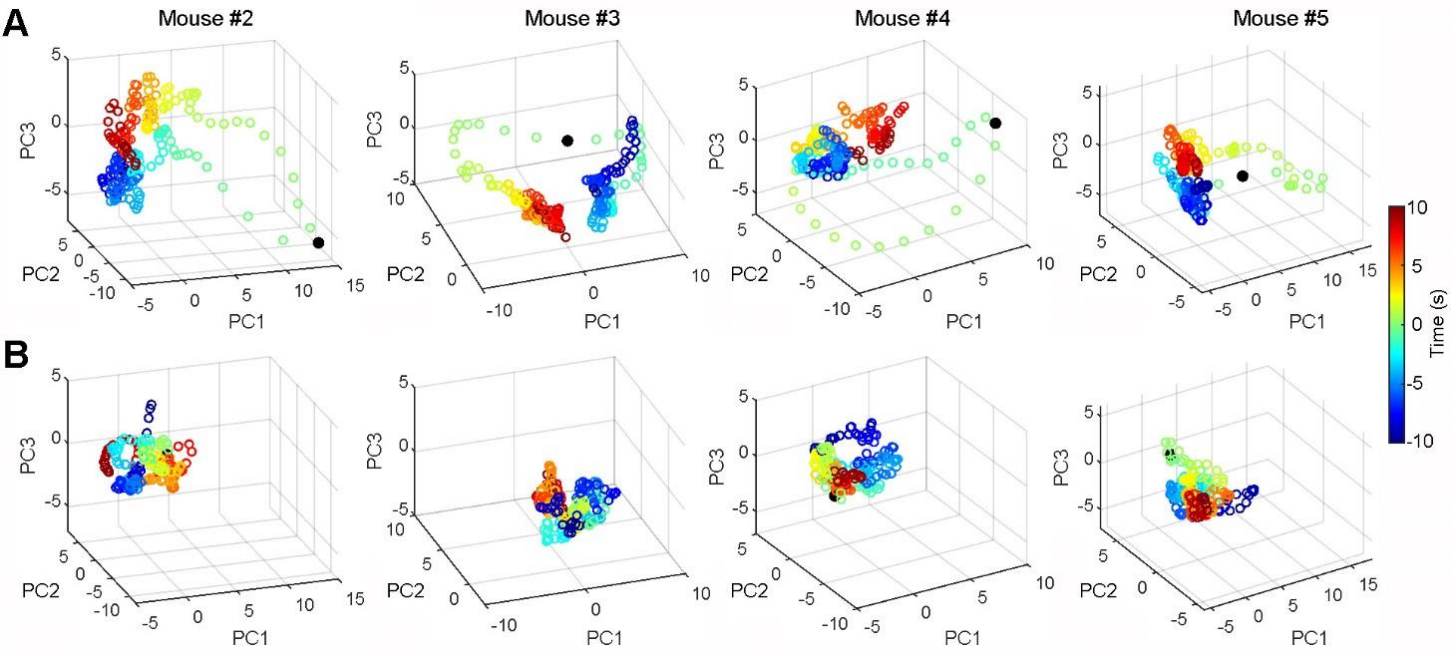
ACC neurons primarily respond during “shuttle” but not “stay” trials. **A&B**, Dimension reduction analysis (i.e., PCA) indicates robust changes in ACC neuronal population activity during correct shuttles (A) but not correct stays (B) from four representative recording sessions. Time “0” indicates shuttle crossings (A) or sound onsets (B), respectively. For more details, see Fig. 2.

Next, we conducted multi-channel *in vivo* recordings from the ACC in mice performing discrimination–avoidance tasks after they achieved a success rate of 70% or above (Fig. 2A). We initially focused on examining how ACC neurons engage in two distinct behavioral responses after the sounds: correct shuttles and correct stays (Fig. 2 B&C). Our results reveal that most ACC neurons exhibit robust changes in activity, either activation or suppression, more often in a sustained manner rather than transiently, primarily after shuttle crossings (Fig. 2 D&E, top panels). In contrast, the same ACC neurons show minimal activity changes during stay trials or in response to auditory cues (Fig. 2 D&E, bottom panels; Supplementary Fig. 3). Further dimension reduction analysis reveals that ACC neuronal population activity patterns drastically change during shuttle trials but remain stable during stay trials (Fig. 2F; Fig. 3). This pronounced ACC activity following shuttles, coupled with minimal activity in response to cues, suggests that ACC neurons primarily encode post-action variables rather than pre-action ones. These extended post-action responses may facilitate complex associative learning by linking temporally separated action- and outcome-relevant information.

To further confirm that ACC activity encodes post-action variable, we examined ACC activity in relation to shuttle initiations. Our results reveal that most ACC neurons (~73%) exhibit sustained activity changes after shuttle initiations (Supplementary Fig. 2; Types 1&2). Notably, the timing of this activation aligns with shuttle crossings rather than shuttle initiations, further suggesting that ACC activity does not encode pre-action variables (Supplementary Fig. 2; Type 1). This finding reinforces the notion that ACC activity primarily encodes post-action variables.

### ACC neurons monitor actions independent of outcomes

**Figure 4.**
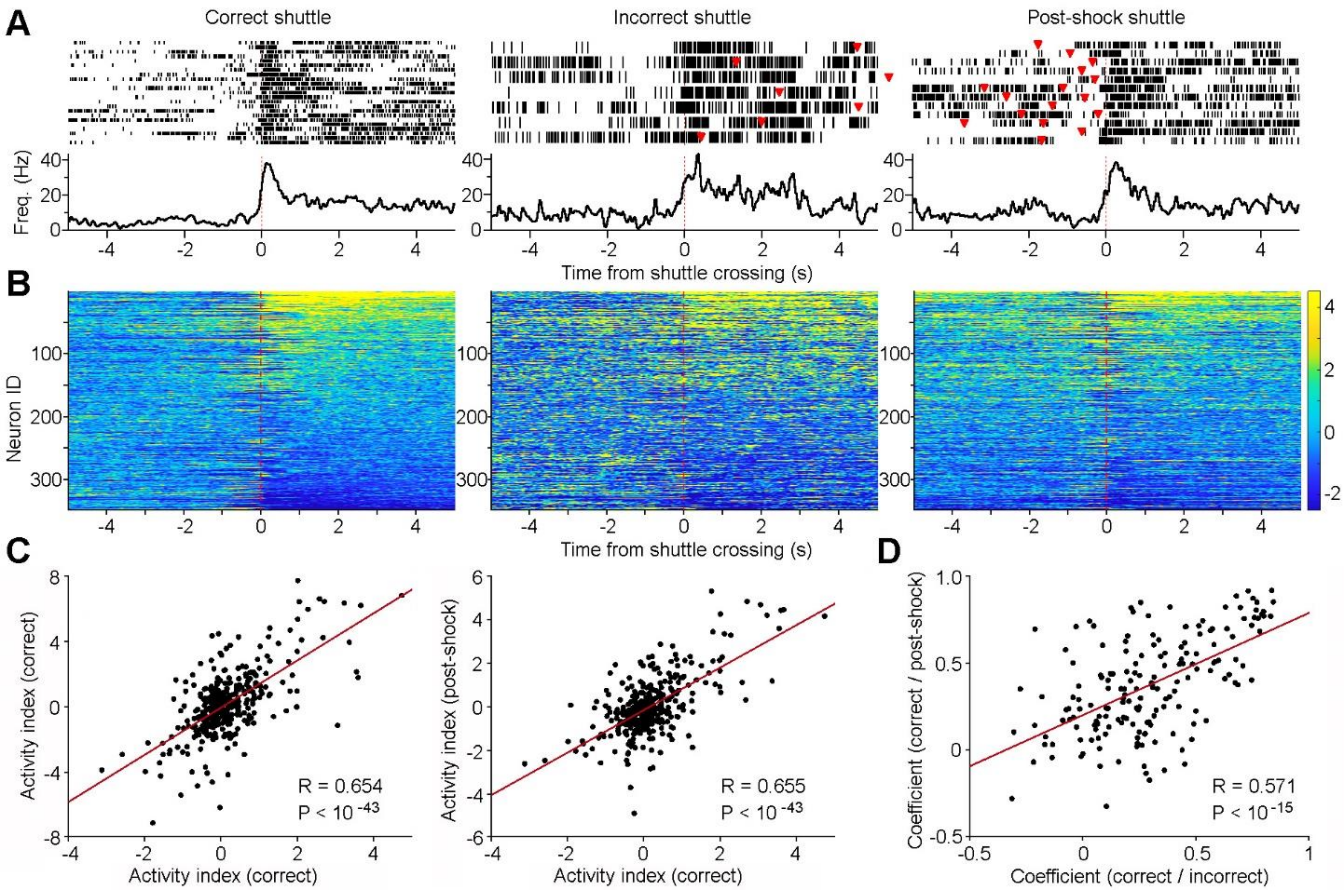
ACC neurons monitor actions independent of outcomes. **A**, Peri-event rasters (trials) and histograms of a representative ACC neuron during correct (left), incorrect (middle), and post-shock shuttles (right) within a session. Red triangles indicate shock administrations. Note that incorrect shuttles are followed by a second shuttle after animals receive footshocks, and approximately half of the post-shock shuttles are preceded by incorrect shuttles. **B**, Heatmaps showing the activity of individual ACC neurons (n = 348) during correct (left), incorrect (middle), and post-shock shuttles (right). Neurons are arranged in the same order across the three heatmaps. Color bar indicates z-scored activity. Note that the number of incorrect shuttles in a session is often ≤7, leading to greater variability in mean activity. Only sessions with ≥4 incorrect shuttles are included in the analysis. **C**, Activity indexes of individual ACC neurons between correct and incorrect shuttles (left), and between correct and post-shock shuttles (right). Activity index is defined as: Activity Index = Mean^post-shuttle^ – Mean^pre-shuttle^, where Mean^pre-shuttle^ and Mean^post-shuttle^ are the mean z scores calculated between −5–0 and 0–5 s, respectively, as shown in B. **D**, Correlation coefficients of the activity (−5–5 s) between correct and incorrect shuttles (x axis) and between correct and post-shock shuttles (y axis). Only the top and bottom quartiles of ACC neurons (as shown in B) are used for the analysis.

To determine if the post-shuttle ACC activity encodes information related to outcomes, we analyzed ACC neuronal activity across three distinct conditions: correct shuttles, incorrect shuttles, and post-shock shuttles (Fig. 4A; post-shock shuttles are defined as shuttles following footshocks received during incorrect-shuttle and incorrect-stay trials). Our results reveal that ACC neurons exhibit similar activity patterns across all three conditions, regardless of outcomes (presumed safety *vs*. uncertainty *vs*. safety; Fig. 4B). Further analysis confirmed significant correlations between these conditions both in activity strength (Fig. 4C) and activity pattern (Fig. 4D). Moreover, ACC neurons show limited responses to footshocks during incorrect trials (Supplementary Fig. 3). Together, these findings suggest that ACC neurons monitor actions independent of outcomes/values associated with these actions.

### ACC neurons monitor *action states* and *action contents*

**Figure 5.**
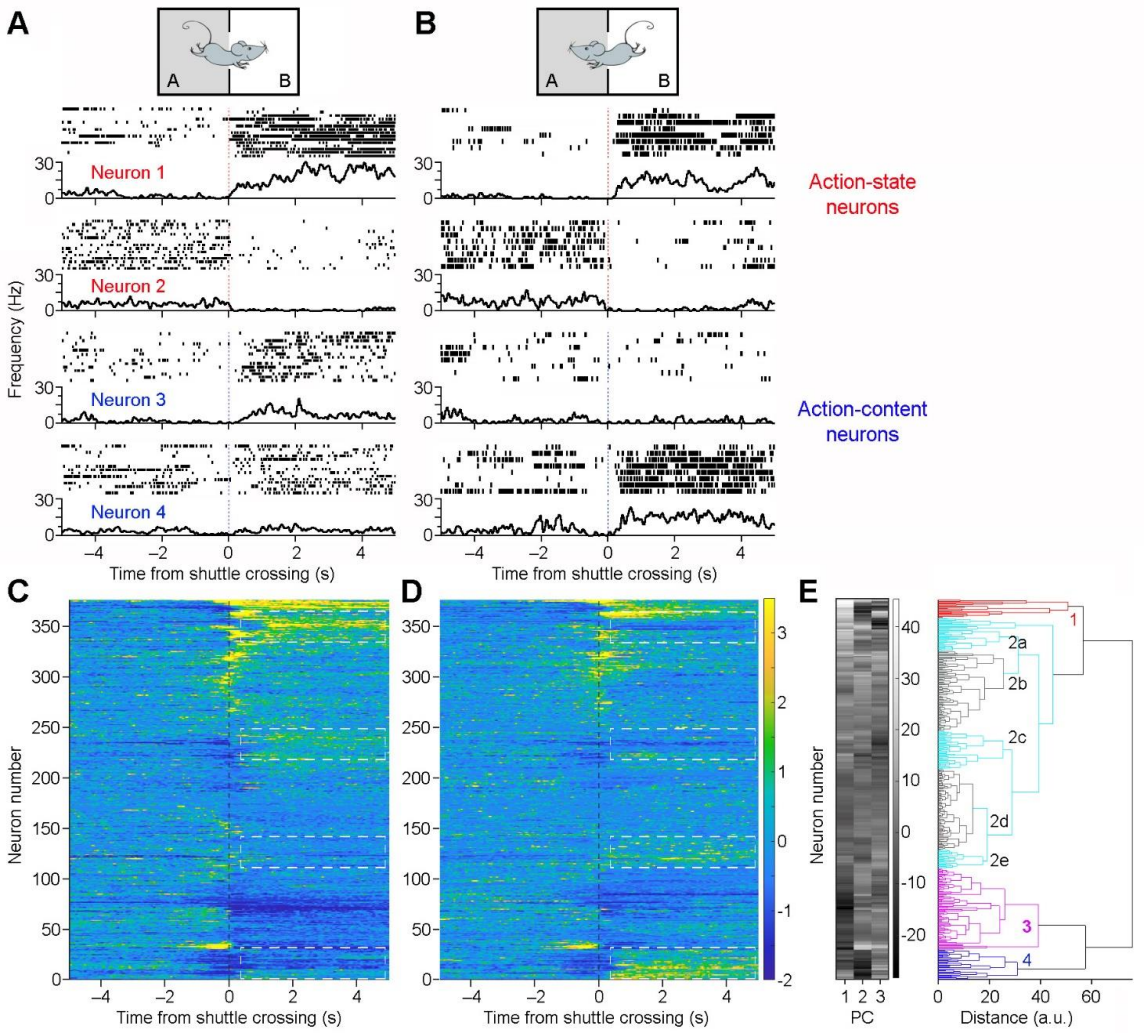
ACC neurons monitor *action states* and *action contents*. **A&B**, Peri-event rasters (trials) & histograms of four simultaneously recorded ACC neurons during two sets of shuttles: rooms A→B shuttles (A) *vs*. rooms B→A shuttles (B). Note that the first two neurons exhibit indiscriminate responses, either increasing (#1) or decreasing their activity (#2) after the shuttles, thereby monitoring action state changes. In contrast, neurons 3&4 are selectively activated in one set of the shuttles, thereby monitoring action contents (i.e., rooms A→B *vs*. B→A shuttles). **C&D**, Z-scored activity of individual ACC neurons (n = 376; recoded from 15 sessions) surrounding rooms A→B shuttles (C) and rooms B→A shuttles (D). Neurons are arranged in the same order in the two heatmaps. **E**, Principal-component analysis (PCA) classifies ACC neurons into four major categories based on their activity changes surrounding shuttle responses. PC1, PC2, and PC3 represent the first three principal components color coded from low (dark) to high scores (white). Note that most neurons in categories 1 and 3 are action-state neurons, whereas most neurons in categories 2a, 2c, 2e, and 4 are action-content neurons.

Next, we investigated which specific post-action variables are encoded by ACC neurons. We categorized correct shuttle responses into two sets: rooms A→B shuttles (in response to sound A) *vs*. rooms B→A shuttles (in response to sound B). Our analyses identified two major groups of ACC neurons based on their responses to these shuttles. The first group shows indiscriminate response, exhibiting either increased activity (Fig. 5: Neuron 1) or decreased activity (Fig. 5: Neuron 2) without differentiating between A→B and B→A shuttles. We propose that these ACC neurons encode an *action state*, a variable representing the change of behavioral state in response to the pair of sounds.

In contrast, the second group of ACC neurons exhibit discriminate responses between rooms A→B and B→A shuttles. These neurons either respond selectively in one set of shuttles or show different levels of responses (activation or inhibition) between the two sets of shuttles (Fig. 5A: Neurons 3&4; Fig. 5 C–E; Supplementary Fig. 4). We propose that these ACC neurons encode *action content*, a variable representing distinct action information (i.e., shuttling from rooms A→B *vs*. B→A). Notably, this shuttle-route response selectivity does not resemble place cell activity observed in the hippocampus [29], as evidenced by our analysis of data from the intertrial-interval periods (Supplementary Fig. 5). This aligns with prior findings that ACC neurons do not simply encode spatial information [30], although they likely incorporate spatial variables.

**Figure 6.**
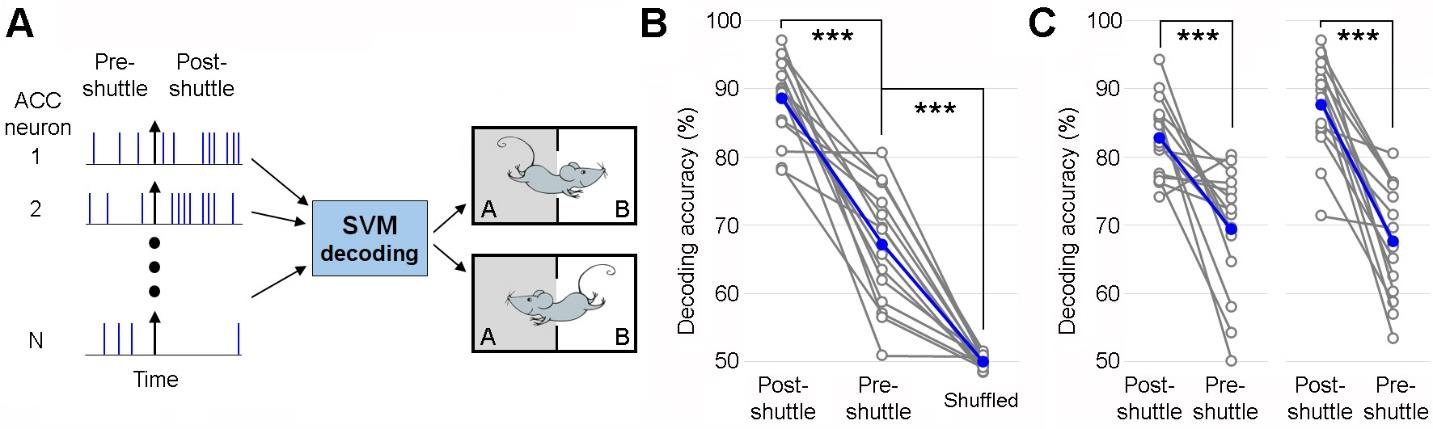
Post-shuttle ACC neuronal population activity decodes *action content*. **C**, Schematic diagram of support vector machine (SVM) decoding. ACC neuronal population activity from pre-shuttle period (−5–0), post-shuttle period (0–5), or shuffled spikes is used to train the decoder and subsequently distinguish between action content (rooms A→B *vs*. B→A shuttles). **B**, Mean decoding accuracy (blue line) and individual decoding accuracies for 15 sessions (grey lines). One-way ANOVA: F_2, 42_ = 146.19, P = 1.2 × 10^–19^. ***P < 0.001, Bonferroni post-hoc test. **C**, The same as B, except that shorter (left, 2.5 s) or longer decoding windows (right, 7.5 s) were used. ***P < 0.001, paired *t* test.

To determine if the post-action ACC neuronal population activity can decode shuttle contents (rooms A→B *vs*. B→A shuttles), we implemented a machine-learning approach. Specifically, we trained binary support vector machine (SVM) classifiers and performed cross-validations (Fig. 6A). Our results revealed that the post-shuttle population ACC activity is highly effective in decoding the animal’s preceding actions of either A→B *vs*. B→A shuttles, reaching a decoding accuracy close to 90% on average (Fig. 6 B&C). Given that action-state neurons show no difference in activity between shuttles, model prediction accuracy is likely derived from activity differences of action-content neurons. Although the pre-shuttle ACC activity can also decode the shuttle content, it does so with much lower accuracy (<70% on average; Fig. 6 B&C). These findings provide compelling evidence supporting the ACC’s involvement in post-action information processing related to the animal’s preceding actions.

### Post-action ACC activity influences future performance

**Figure 7.**
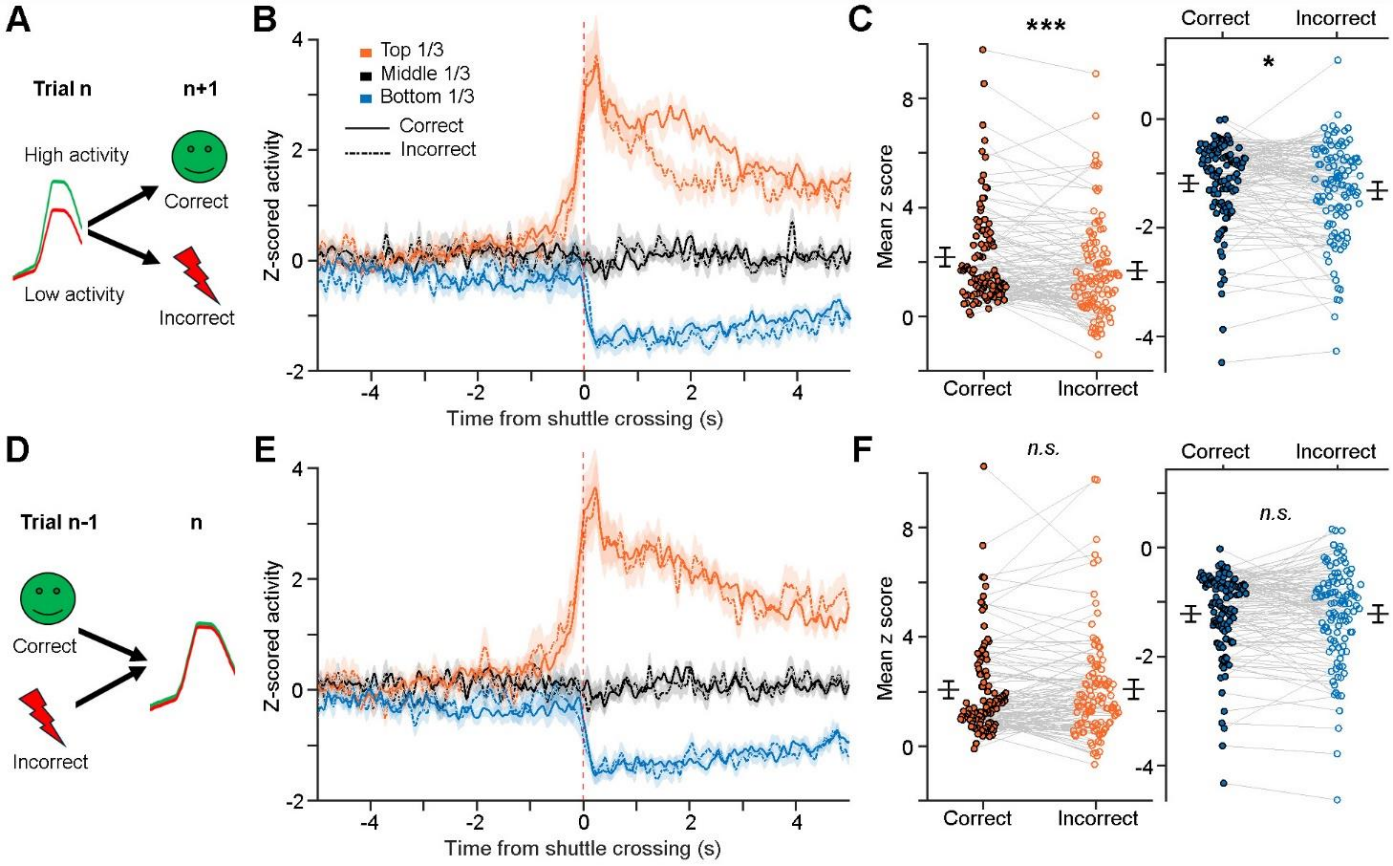
Post-action ACC activity influences future performance within a task session. **A**, Schematic illustration. **B**, Mean activity (± s.e.m.) of post-shuttle activated neurons (orange lines; top 1/3), inhibited neurons (blue lines; bottom 1/3), and remaining ACC neurons (black lines; middle 1/3), which preceded either correct trials (solid lines) or incorrect trials (dashed lines). **C**, Further comparison of the activation strength for post-shuttle activated neurons (left) and inhibited neurons (right) between the two conditions. Each pair of dots indicates an ACC neuron. **D–F**, Similar to A–C, except that the comparison is based on the status of the preceding trials. Mean z scores in C and F were calculated between 0–5 s after shuttle crossings. Paired *t* test, n.s., non-significant; *P < 0.05; ***P < 0.001.

We next investigated whether post-action ACC activity plays a role in subsequent-trial performances. Specifically, we divided correct-shuttle trials into two groups (Fig. 7A): one followed by correct trials (including both correct stays and shuttles), and the other followed by incorrect trials (including incorrect stays and shuttles). Our results reveal that post-shuttle ACC activity is notably higher when it preceded correct trials rather than incorrect ones (Fig. 7B). Statistically, both the top and bottom thirds of the most responsive ACC neurons exhibit significantly higher activity that preceded correct trials than incorrect ones (Fig 7C). This correlation between higher ACC activity and future correct performance suggests that post-action ACC activity may contribute to trial-to-trial behavioral adjustment underlying associative learning [31].

As a control, we also examined whether the status of the preceding trial influences post-action ACC activity in the current trial. Specifically, we divided correct-shuttle trials into two groups: one preceded by correct trials and the other preceded by incorrect ones (Fig. 7D). Our results show no difference in post-shuttle ACC activity between the two conditions (Fig. 7 E&F). This finding is expected, as both positive and negative reinforcement can similarly contribute to associative learning and neural plasticity.

## Discussion

For decades, researchers have explored the role of the ACC in cognitive and adaptive behavioral processes. This body of work has led to a series of contentious findings, with the ACC being implicated in a range of functions, including error detection, conflict monitoring, action valuation, outcome valuation, attention, decision-making, and strategy updating [8–28, 30–41]. More recent findings challenge these interpretations, suggesting that these roles reflect features of the ACC’s involvement in cognitive processes rather than its core function [1–7]. The lack of consensus, and at times contradictory findings, may stem from inconsistencies in task structure across ACC research. In particular, the often lack of clear temporal boundaries between actions and outcomes complicates interpretation. Moreover, much of the research has been conducted in primates and humans, often relying on imaging techniques like fMRI that offer limited temporal resolution of neural activity. Additionally, the homology of ACC functions between primates and rodents remains a subject of debate [42]. As a result, an inconclusive narrative about the ACC’s role persists.

Here we developed a novel discrimination–avoidance task in combination with electrophysiology recordings for mice. This task offers a more sophisticated approach to investigate associative learning, as performance depends not only on sensory and contextual discrimination, but also on linking specific cues, actions, and outcomes. Unlike the conventional Pavlovian conditioning model, which associates one stimulus with a single outcome, our task involves each stimulus (sound) leading to one of two possible outcomes (shock or no shock), depending on the context. Therefore, a simple Pavlovian association strategy is unlikely to be sufficient for learning the task. Additionally, our discrimination–avoidance task is physically demanding, requiring shuttle responses from room to room, which promotes the investigation of more natural action responses. Finally, this procedure allows us to compare information coding at critical time windows surrounding action responses (shuttle or stay) and outcome deliveries (shock or no shock) with high temporal resolution. Overall, we find that mice can learn and perform the task with high proficiency, and that this performance is maintained during electrophysiology recordings.

Utilizing the discrimination–avoidance task, we find that the ACC primarily encodes post-action variables. Specifically, ACC neurons exhibit robust post-shuttle responses across various conditions, including correct, incorrect, and post-shock shuttles. Despite these conditions signaling distinct outcomes (presumed safety *vs*. uncertainty *vs*. safety), the response properties of the ACC neurons remain consistent. Moreover, very few ACC neurons respond directly to positive outcomes (safety) or negative outcomes (shocks). These findings suggest that, in our task, ACC activity primarily monitors animal’s most recent actions (i.e., action states and action contents) rather than the outcomes or values associated with those actions [22, 27, 28].

Our results further reveal two distinct groups of ACC neurons that encode different aspects of actions: *action state* and *action content*. Action states appear to represent the animal’s immediate choice of whether an action was taken, updating changes in behavioral state within the ongoing task. In contrast, action content represents the specific actions taken (i.e., shuttles from rooms A→B *vs*. B→A), preserving detailed action information. The response selectivity of *action content*-encoding ACC neurons further supports the notion that ACC activity monitors preceding actions rather than action-associated outcomes or values, because both the outcomes and values of these actions are uniform. Notably, our findings do not support an alternative interpretation that *action content* reflects correct *vs*. incorrect shuttles. Theoretically, categorizing actions solely as correct or incorrect may not be meaningful for the brain, as both positive and negative outcomes can guide future actions and contribute to learning [28].

Our study also reveals that ACC neurons play a minor role in encoding pre-action variables, as evidenced by their limited responses to auditory cues and minimal activity changes prior to shuttle initiations during discrimination–avoidance tasks. Consistently, our machine learning decoding shows that the pre-action ACC activity has much less predictive power on animals’ behaviors compared to post-action ACC activity. These findings align with recent research suggesting that ACC neurons are mainly involved in post-decisional information processing rather than decision-making itself [19–21]. Nevertheless, substantial evidence from other studies supports the ACC’s contribution to decision-making, as indicated by its differential responses to discriminative sensory cues preceding decisions [32, 41, 43]. Several factors may explain the discrepancy in our findings, including the unique nature of our task and limitations in our study, which are discussed below.

A key distinction is that the sensory cues in our task are not intrinsically linked to specific values. Although these cues play a crucial role in driving go (shuttle)/no-go (stay) decisions, their values are context-dependent and vary across trials. This dynamic nature of value association may explain why we observe minimal response of ACC neurons to these cues. In contrast, the ACC activity reported in previous studies likely reflects the stable value of the cues, which is important for optimizing future performance but not necessarily essential for decision-making itself [40, 44].

One caveat of our study is that the discrimination–avoidance task requires weeks of training in mice. By the time they master the task, ACC activity likely reflects modified neural circuits. Investigating ACC activity during early phase of learning, such as by introducing a new pair of cues or contexts, could provide further insights into the ACC’s role in learning and cognitive processes. Additionally, previous studies have highlighted the ACC’s key role in reversal learning [45–48]. Future research examining how ACC neurons respond when task rules are reversed could further illuminate this area. Finally, our discrimination–avoidance task revolves around one specific action: shuttling. Future studies are needed to determine whether ACC action monitoring extends to other behaviors. Given recent research highlighting the ACC’s role in post-decisional information processing, post-action ACC activity is likely broad and not limited to shuttling [19–21].

Lastly, our findings suggest that post-action ACC activity plays a key role in shaping future behavior. Specifically, we find that increased post-action ACC activity is linked to future performance in subsequent trials, highlighting the ACC’s role in facilitating learning and guiding behavior. This aligns with previous research demonstrating the ACC’s critical role in complex and flexible learning, such as discriminative avoidance learning [49], discriminative extinction learning [50], reversal learning [45–48], task switching [41], trial-to-trial behavioral adaptation [31], and action–outcome associative learning [14, 27, 28].

Taken together, we speculate that both *action state*-encoding and *action content*-encoding ACC neurons contribute to complex associative learning through extended signaling of action-relevant information, thereby bridging cue, action, and outcome associations. Specifically, the *action state*-encoding ACC neurons signal whether an action was taken, while the *action content*-encoding ACC neurons provide specific details about what the action was – the content. This action-related information, eventually, is integrated with cue- and outcome-related variables to form complex cue–action–outcome associations that guide future behavior. Given our key finding that the ACC primarily encodes action-related variables rather than cue- or outcome-related information, we speculate that the integration of these complex associations takes place in other higher cognitive areas, such as the prelimbic area of the prefrontal cortex (Supplementary Fig. 6) [51–53].

## Methods

### Mice.

Male C57BL/6 mice (Jackson Laboratory, stock #000664) were used in this study. The mice were 8–10 weeks old at the start of discrimination–avoidance task training. All mice were group-housed (2–4 mice per cage; 40 × 20 × 25 cm) with corn cob bedding and cotton nesting material. They were maintained on a 12-h light/dark cycle with *ad libitum* access to food and water. All experimental procedures were approved by the Institutional Animal Care and Use Committees and adhered to the National Research Council’s Guide for the Care and Use of Laboratory Animals.

### Sounds and shuttle box.

Two 10-s auditory cues, 5-kHz tone at ~75 dB and white noise at ~65 dB, were chosen for sound discrimination. Both sounds included 50-ms shaped rise and fall times to reduce abruptness and minimize potential startle responses. The shuttle box used in the experiment was a square chamber measuring 25 × 25 × 32 cm, with a 36-bar shock grid floor, illuminated by lights inside sound-attenuating cubicles (64 × 75 × 36 cm) equipped with speakers (*Med Associates*). The shuttle box was divided at the midline by a plastic divider into two rooms. These two rooms were slightly modified for discrimination purposes: one had two black walls plus two other original walls, while the other room had one white wall plus three original walls (Supplementary Fig. 1). The divider had a 2-inch opening in the center to allow the mice to move freely between the rooms. Animals’ behaviors were recorded using Video Freeze software (*Med Associates*) [54].

### Discrimination–avoidance task.

Prior to training, all mice underwent two daily handling sessions (~10 min each). Once training began, the mice received one training session per day, 5 days per week. In the task, the mice were trained to discriminate between two distinct sounds (A and B) and shuttle between two adjacent rooms (A and B) within a shuttle box to avoid potential footshocks. Specifically, sounds A and B signaled electric shocks in rooms A and B, respectively (Fig. 1A). During training, the mice were first allowed to freely explore the shuttle box for 2 min before trials began, except on the first day of training, where the free exploration period was extended to 10 min.

The training procedure consisted of two phases: pre-training and training. Pre-training phase: mice underwent five daily sessions of sound alternation trials 5/5 (AAAAABBBBB …). Mice that did not exhibit shuttling responses during the first session were excluded from further training. Training phase: mice underwent 10 daily sessions of alternation trials in a pseudorandom order (ABBABAAB …).

Each training session comprised 50 trials, 60 s apart. On incorrect trials, up to 10 scrambled electric shocks (0.5 mA; 0.1 s) were administered starting at sound terminations and continued for an additional 18 s (1 shock every 2 s), except during the pre-training phase, where up to 20 scrambled electric shocks were administered. Shocks were terminated once the mice navigated to the adjacent safe room. The mouse’s success rate in avoiding shocks at sound terminations was defined as: Success rate (%) = (Correct stays + Correct shuttles) / All trials.

### Real-time location detection.

We employed MATLAB functions to detect animal location and subsequently control shock administration (Supplementary Fig. 1). Specifically, *Med Associates* Video Freeze software recorded real-time video footage of the animal’s behaviors [54]. During each trial, MATLAB captured screenshots of the ongoing video at sound terminations and every 2 s thereafter during the shock period. MATLAB then performed background extraction on the captured images to determine which room the mouse was located in. To deliver a shock, MATLAB sent a “shock” signal to an *Arduino UNO* circuit board, which relayed that signal to *Med Associates*, triggering shock administration.

### Stereotaxic surgery.

Mice that surpassed a success rate of 70% during discrimination–avoidance tasks were used for surgery. In brief, mice were anesthetized with ketamine/xylazine mixture (~100/10 mg/kg, i.p.) and maintained on a heating pad at 37°C. Following that, mice received an intra-ACC implantation of a custom-made electrode array (8 tetrodes) [55, 56], and the implant was secured to the skull with stainless screws and dental cement. The coordinates used were AP 1.0 mm, ML 0.4 mm, and DV 1.0 mm.

### *In vivo* recording during discrimination–avoidance tasks.

We used tetrodes for recording. Each tetrode consisted of four wires (90% platinum 10% iridium; ~18 μm diameter; *California Fine Wire*). Neural signal was preamplified, digitized, and recorded using a *Blackrock Neurotech* CerePlex, while the animals’ behaviors were simultaneously recorded. Spikes were digitized at 30 kHz and filtered between 600–6000 Hz. The recorded spikes were manually sorted using *Plexon* Offline Sorter, with key datasets verified by a second experimenter. Tetrode arrays were gradually lowered through a microdrive [57, 58] to record at various depths within the ACC, with 2–4 depths from each animal (DV ~1.0–1.3) used for analysis. Overall, ACC spikes from five mice across 15 sessions were analyzed, with neuron counts of 20/23/29/32, 33/25/21/27, 17/20/22, 28/24, and 22/25, respectively.

The discrimination–avoidance tasks were similar to that during the training phase, except that five direct-current electric shocks, which minimize electromagnetic artifacts, were administered starting at sound terminations and continued for 8 s. This shorter shock period allowed the mice more time to move freely within the shuttle box during the 42-s inter-trial intervals, when no consequences were administered.

### Shuttle behavior analysis.

We used DeepLabCut [59] to analyze animals’ locations during discrimination–avoidance tasks, with all locations determined based on body center positions. Shuttle crossing was defined as body center crossing the midline opening of the shuttle box. Shuttle initiations and terminations were defined as the time points when animal’s movement velocity deviated 1 s.d. above mean (Supplementary Fig. 2A).

### Dimension reduction.

We used Principal Component Analysis (PCA) to analyze the major activity patterns of ACC neuronal populations. The first three principal components (PCs) were used for 3-D visualizations. Specifically, the activity of each ACC neuron was first averaged during shuttle responses (bin size, 0.1 s) and z scored. The z-scored activity of ACC neurons was then processed with PCA (*pca*, MATLAB).

### Classification of ACC neuronal types.

We used PCA to classify major types of ACC neurons based on their activity surrounding rooms A→B and B→A shuttles. Specifically, the activity of each ACC neuron was first averaged surrounding shuttle responses (−5–5 s; bin size, 0.25 s) and z-scored. The first three PCs were used in a hierarchical clustering algorithm (Linkage) to find the similarity (Euclidean distance) between all pairs of activity patterns in PC space, iteratively grouping the activity patterns into larger and larger clusters based on their similarity. Lastly, we set a distance-criterion to extract major clusters from the hierarchical tree [60].

### Machine learning decoding.

We used Support Vector Machine (SVM) classifiers to train ACC neuronal population activity to decode shuttle content (rooms A→B *vs*. B→A shuttles) and subsequently performed cross-validations. Specifically, the total number of spikes from each ACC neuron, calculated during either the pre-shuttle (−5–0 s) or post-shuttle period (0–5 s), were used for training and testing the SVM classifier. For each dataset, we performed SVM classification training and cross-validation 100 times (*fitcsvm* and *crossval*, MATLAB), assigning the mean correct classification rate as the decoding accuracy. Notably, using shorter or longer time windows surrounding shuttle responses, such as 2.5 or 7.5 s, yielded similar conclusions.

### Histology.

To mark the final recording sites, we made electrical lesions by passing 10-s, 10-μA currents through multiple tetrodes. Mice were deeply anesthetized and intracardially perfused with ice-cold PBS or saline, followed by 10% formalin. The brains were removed and postfixed in formalin for at least 24 hours. The brains were sliced into coronal sections of 50-μm thickness using *Leica* vibratome. Brain sections were mounted with Mowiol mounting medium for microscopic examination of electrode array placements.

### Statistics.

Sample sizes were based on previous similar studies. Statistical analyses include Analysis of Variance (ANOVA) followed by post-hoc Bonferroni, and Student’s *t* test. All statistical tests are two-sided; P-values of 0.05 or lower were considered significant.

## Supplementary Material

1
