# Supplementary material for: Anterior cingulate cortex in complex associative learning: monitoring action state and action content
Source: bioRxiv. 2025 Jan 29:2025.01.29.635442. Preprint. [Version 1] doi: 10.1101/2025.01.29.635442 (PMC11838375; doi:10.1101/2025.01.29.635442)
Supplement: 1 [file NIHPP2025.01.29.635442V1-supplement-1.pdf]

bioRxiv preprint doi: https://doi.org/10.1101/2025.01.29.635442; this version posted January 29, 2025. The copyright holder for this preprint (which was not certified by peer review) is the author/funder, who has granted bioRxiv a license to display the preprint in perpetuity. It is made available under aCC-BY 4.0 International license.

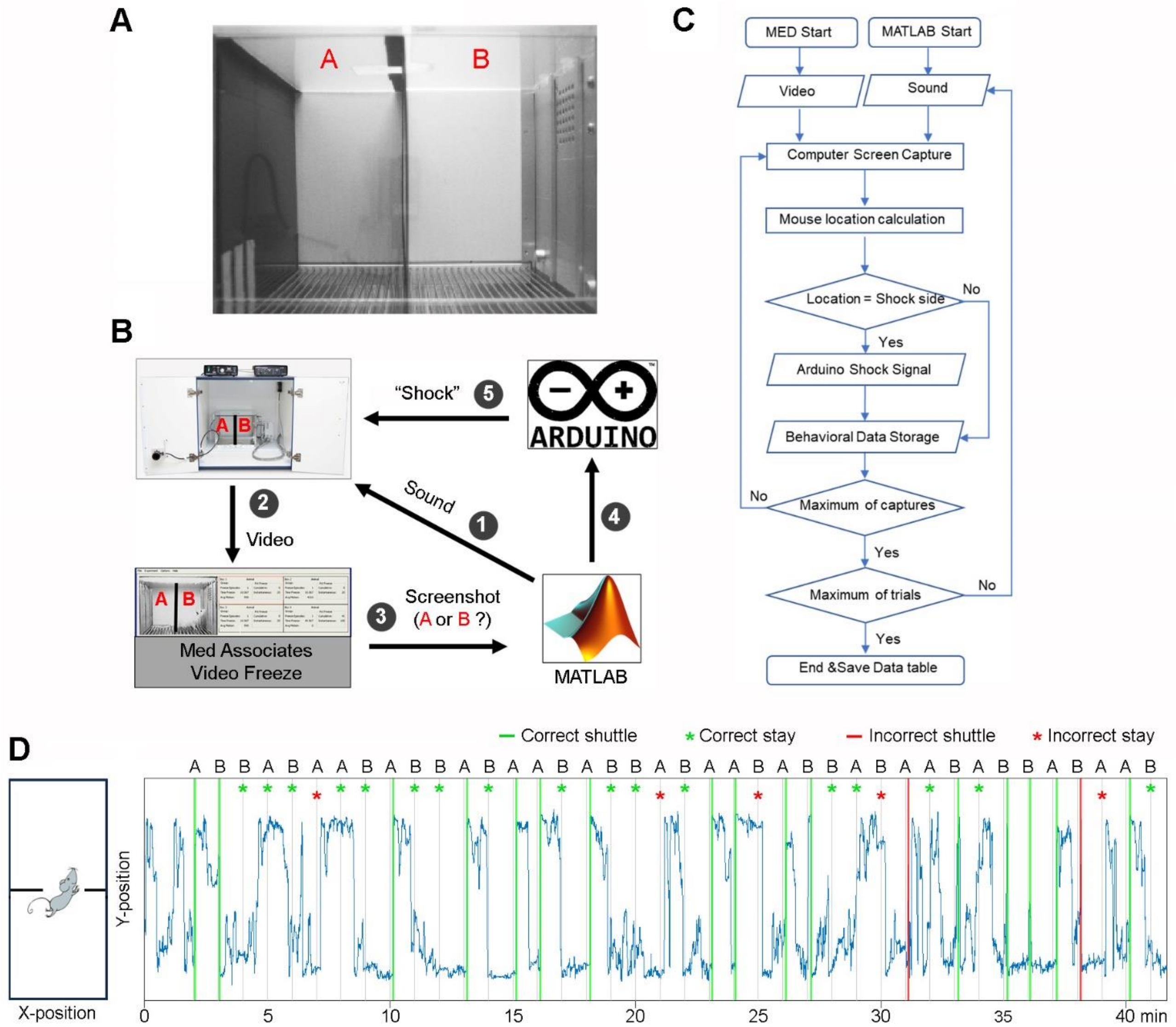

Supplementary Fig. 1. Experimental setup. A, The shuttle box used for behavioral training. Room A is configured with two black walls (left and right), one white wall (back), and a transparent front wall for video recording purposes. Room B is configured with two white walls (left and back), one metal wall (right), and a transparent front wall. **B,** Schematic diagram of the control setup utilizing MATLAB functions, with numbers indicating the sequence of control flow. **C,** A comprehensive flowchart illustrating the control setup as shown in B. **D,** Left, sounds A and B signal shocks in the bottom and top rooms of the shuttle box, respectively. Right, the Y position of a well-trained mouse in a ~40-min session.

bioRxiv preprint doi: https://doi.org/10.1101/2025.01.29.635442; this version posted January 29, 2025. The copyright holder for this preprint (which was not certified by peer review) is the author/funder, who has granted bioRxiv a license to display the preprint in perpetuity. It is made available under aCC-BY 4.0 International license.

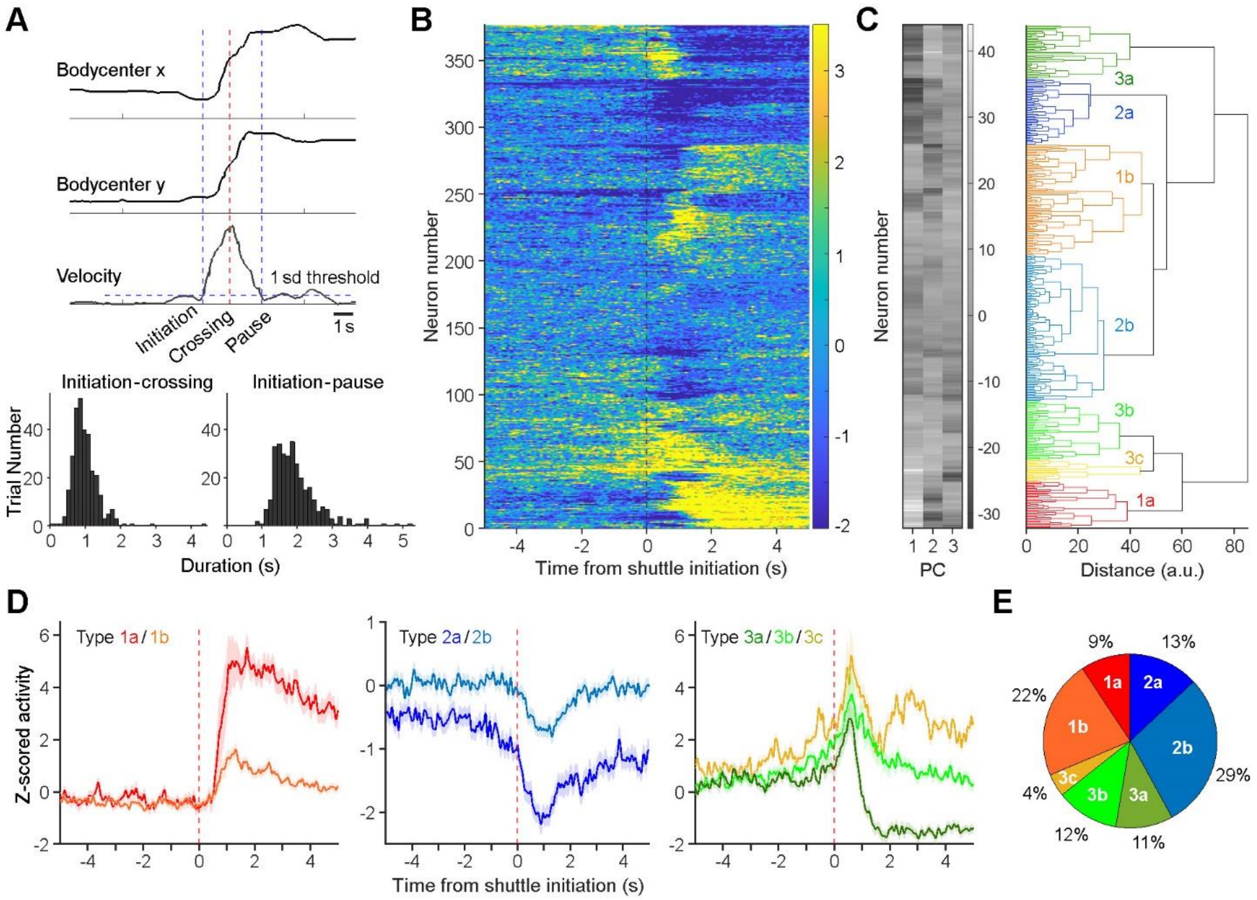

Supplementary Fig. 2. Characterizing ACC neuronal activity in relation to action initiations. A, Top, schematic illustrating the definition of shuttle initiation, crossing, and termination (pause). Bottom, distributions of half- (left) and full-shuttle durations (right). **B**, Z-scored activity of all ACC neurons during correct shuttle trials. **C,** Principal-component analysis (PCA) classifies ACC neuronal activity (as shown in B) into seven categories. PC1, PC2, and PC3 represent the first three principal components color coded from low (dark) to high scores (white). **D**, Mean activity (± s.e.m.) of the seven categories of ACC neurons. **E**, Fractions of individual categories of ACC neurons.

bioRxiv preprint doi: https://doi.org/10.1101/2025.01.29.635442; this version posted January 29, 2025. The copyright holder for this preprint (which was not certified by peer review) is the author/funder, who has granted bioRxiv a license to display the preprint in perpetuity. It is made available under aCC-BY 4.0 International license.

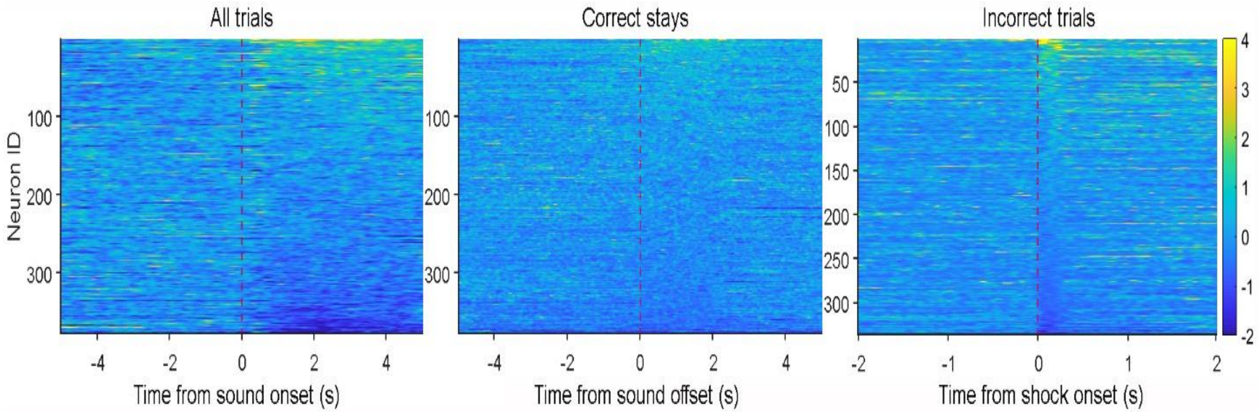

Supplementary Fig. 3. ACC shows limited response to cue, stay trials, and footshocks. Left**,** Heatmap showing the activity of individual ACC neurons (n = 348) in relation to auditory cue onset. **Middle,** Heatmap showing the activity of individual ACC neurons (n = 348) during stay trials. **C,** Heatmap showing the activity of individual ACC neurons (n = 336) during footshock. Note, footshock trials with a shuttle response within 1 s shock onset were excluded to avoid shuttle response confound. As a result, some behavioral sessions were excluded due to insufficient trial numbers.

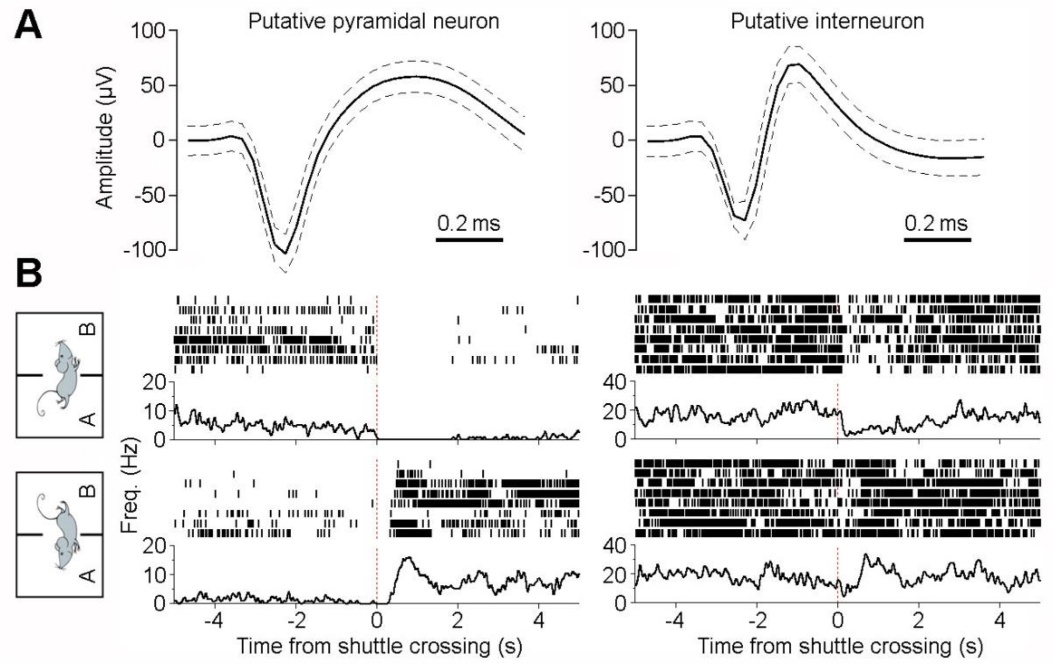

Supplementary Fig. 4. ACC pyramidal neurons and interneurons both monitor action content. **A,** Spike waveforms (mean ± s.d.) of two representative ACC neurons: one putative pyramidal neuron and one interneuron. These two neurons were recorded simultaneously. **B,** Peri-event rasters (trials) & histograms of the same two ACC neurons surrounding shuttle responses. Both neurons exhibit differential activity changes that discriminate between rooms A→B (top panels) *vs.* B→A shuttles (bottom panels).

bioRxiv preprint doi: https://doi.org/10.1101/2025.01.29.635442; this version posted January 29, 2025. The copyright holder for this preprint (which was not certified by peer review) is the author/funder, who has granted bioRxiv a license to display the preprint in perpetuity. It is made available under aCC-BY 4.0 International license.

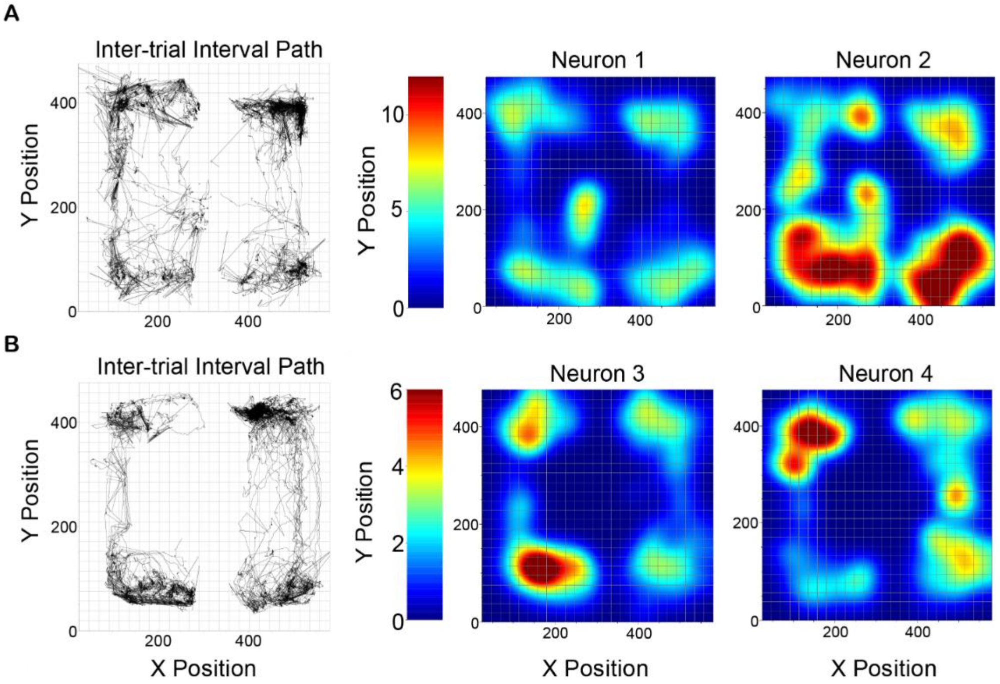

587

588 **Supplementary Fig. 5. ACC neurons do not display place cell activity characteristics. A,** Left, a
589 representative path throughout all inter-trial intervals during a training session, excluding data within 5 s of
590 any shuttle responses. Middle and right, place field activity of two representative ACC action-content
591 neurons. Both neurons show spiking activity across the chamber without place preference. The color bar
592 indicates spikes/s. **B,** Similar to A but from a different animal. Note that these neurons are the same
593 neurons depicted in Figure 5 (#3 & #4).

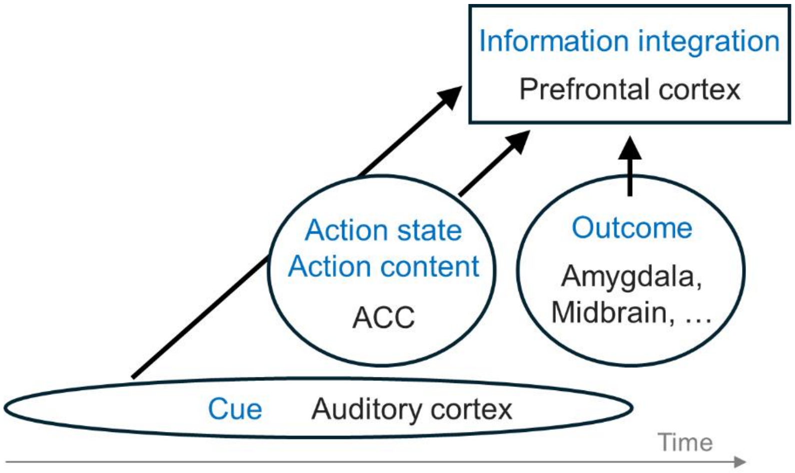

594

595 **Supplementary Fig. 6. A simplified model of information integration during complex cue–action–**
596 **outcome associative learning.** The integration occurs around the time of outcome delivery, when all
597 three sets of information (cue, action, and outcome) are available.
